# Quality of life, household income, and dietary habits are associated with the risk of sarcopenia among the Chinese elderly

**DOI:** 10.1007/s40520-023-02656-9

**Published:** 2024-02-09

**Authors:** Hua Wan, Yan-Hui Hu, Wei-Peng Li, Quan Wang, Hong Su, Jun-Yan Chenshu, Xiang Lu, Wei Gao

**Affiliations:** 1 Department of Geriatrics, School of Medicine, Zhongda Hospital, Southeast University, No.87 Dingjiaqiao, Nanjing, 210009 Jiangsu China; 2 Department of Geriatrics, Sir Run Run Hospital, Nanjing Medical University, No.109 Longmian Avenue, Nanjing, 211166 China; 3 Department of Health Management, Sir Run Run Hospital, Nanjing Medical University, Nanjing, China; 4 Department of Public Health, Sir Run Run Hospital, Nanjing Medical University, Nanjing, China

**Keywords:** Sarcopenia, Health-related quality of life, Household income, Lifestyle, Elderly

## Abstract

**Background:**

Health-related quality of life (HRQoL), which can be influenced by various aspects, especially socioeconomic status and lifestyle, has been identified as an important predictor of the prognosis of older adults. Dietary habit, a major part of lifestyle, can affect the nutritional status, which is closely correlated with the development of geriatric syndromes in the elderly.

**Aims:**

The aim of the study was to examine the association of HRQoL, socioeconomic status, and lifestyle with the risk and severity of sarcopenia, a geriatric syndrome characterized by progressive loss of skeletal muscle mass, strength and function.

**Methods:**

A cross-sectional retrospective study with 2877 participants aged ≥65 years was performed. HRQoL was assessed using EuroQoL Five Dimensions questionnaire. Socioeconomic status was assessed by the educational attainment, occupation, and household income. Lifestyle was assessed using 12 items closely related to Chinese living habits. The information of daily dietary habits including tea, alcohol, type of diet, and volume of drinking water were collected. The associations of HRQoL, socioeconomic status, and lifestyle with the risk of sarcopenia were examined by multivariate regression logistical analysis. The potential causal role of age, body mass index, and waist circumference in the effect of HRQoL on sarcopenia risk was analyzed by causal mediation analysis.

**Results:**

High HRQoL [adjusted odds ratio (OR) =0.85, 95% confidence interval (CI) =0.69–0.95, *P*=0.034] and household income levels (adjusted OR =0.74, 95% CI =0.57–0.95, *P*=0.019) were inversely associated with the risk of sarcopenia. Meanwhile, more consumption of spicy food (adjusted OR =1.34, 95% CI =1.09–1.81, *P* =0.037) and occasionally drinking (adjusted OR =1.46, 95% CI =1.07–2.00, *P* =0.016, as compared to those never drinking) were associated with higher risk of sarcopenia, while skipping breakfast occasionally (adjusted OR =0.37, 95% CI =0.21–0.64, *P* <0.001, as compared to those eating breakfast every day) and less consumption of salt (adjusted OR =0.71, 95% CI =0.52–0.96, *P* =0.026, as compared to those consuming high amount of salt) were associated with lower risk of sarcopenia. Further causal mediation analysis aimed to explore how much age, body mass index, and waist circumference might explain the effect of HRQoL on the risk of sarcopenia showed that the estimated proportion that mediated the effect of HRQoL on the risk of sarcopenia by age was 28.0%.

**Conclusions:**

In summary, our findings demonstrate that low levels of HRQoL and household income, more intake of salt and spicy food, and occasional intake of alcohol are correlated with higher risk of sarcopenia, while skipping breakfast occasionally is associated with lower risk of sarcopenia in a Chinese population of older adults.

**Supplementary Information:**

The online version contains supplementary material available at 10.1007/s40520-023-02656-9.

## Introduction

Sarcopenia is a geriatric syndrome characterized by progressive loss of skeletal muscle mass, strength, and function [1]. According to the China Health and Retirement Longitudinal Study (CHARLS), the prevalence of possible sarcopenia, sarcopenia, and severe sarcopenia was 38.5%, 18.6%, and 8.0%, respectively [2]. . Emerging evidence has linked sarcopenia with poor quality of life, which related to increased likelihood of adverse outcomes including falls, fractures, physical disability, and morbidity, especially in the older adults [1]. It is, therefore, important to screen subjects with high risk of sarcopenia in the elderly.

Health-related quality of life (HRQoL) is a broad concept incorporating subjective symptoms, functioning in multiple life domains, as well as the satisfaction of disease control [3]. Current evidence has suggested poor HRQoL as one of the significant predictors of adverse clinical outcomes including disability, hospitalization, and mortality [4]. Reports on HRQoL in the elderly have revealed an association of quality of life with physical performance such as handgrip strength and gait speed, both of which reflect the function of skeletal muscle, indicating a potential predictive effect of HRQoL on the risk of sarcopenia [5–7]. HRQoL can be affected by socioeconomic status, which is also one of the substantial factors of healthy challenges in the elderly [8]. The Global Burden of Disease Report highlighted the strong association between human health and sociodemographic indices such as total income per capita and educational attainment [9]. Previous study found that lower socioeconomic status was correlated with higher prevalence of frailty, hospitalization, and mortality in the elderly [8, 10, 11]. Interestingly, there is also a significant association of low socioeconomic status with increased risk of sarcopenia [12–14]. Another recent study found that the development of sarcopenia was related to lifetime occupation in the elderly, with blue-collar workers being most affected by sarcopenia and white-collar workers being most affected by sarcopenic obesity [15]. It is worth noting that the provision of lifestyle such as physical activity [16], smoking habits [17], and type of dietary intake [18, 19] also has great influence on the status of HRQoL, regardless of socioeconomic status. Indeed, decreased physical activity, smoking, excessive alcohol drinking, and insufficient nutrient intakes have been reported to be essential factors for the lifestyle changes that lead to sarcopenia [20]. Although there has been plenty evidence showing that poor HRQoL is associated with sarcopenia; however, the exact causal effect of poor HRQoL on sarcopenia remains controversial [21]. Considering that HRQoL, which can be influenced by socioeconomic status and lifestyle, has been identified as an important predictor of the prognosis in the older adults, we therefore aimed to investigate the association of HRQoL with the risk and severity of sarcopenia, as well as the potential interactions of socioeconomic status and lifestyle with HRQoL in the correlation between HRQoL and sarcopenia in a community-dwelling Chinese population of older adults.

## Methods

### Study population

This study was based on the National Basic Public Health Project, which provides annual physical examinations for the old adults in China. Data were extracted from the individuals aged ≥65 years who participated in the project in Yuetang Community Medical Center in Yangzhou, Jiangsu Province in 2020 as previously described [22]. Participants with the following conditions were excluded: (1) unable to move independently or unable to complete the specified actions; (2) severe cardiopulmonary insufficiency (New York Heart Association heart failure was classified as grade III and IV or unable to withstand the 6-meter walking test); (3) severe renal insufficiency (creatinine clearance rate <60mL/min) or severe liver damage (levels of transaminase greater than twice the normal values); and (4) malignant tumor. This study was performed in accordance with the principles outlined in the Declaration of Helsinki [23] and approved by the Ethics Committee of Sir Run Run Hospital, Nanjing Medical University (approval number 2019-SR-S041). Written informed consent was obtained from each participant.

### Data collection

Demographic variables which included marriage status, residency, income sources, payment methods for medical expenses were collected from the participants by self-report. Waist circumference was measured and body mass index was calculated by dividing weight by height squared. For blood and biochemical parameters, venous blood sample was collected in the early morning after an overnight fasting and separated into serum and cellular fractions within 2 hours. The serum was stored at -80 °C before further analysis. Total cholesterol, triglycerides, high-density lipoprotein cholesterol, low-density lipoprotein cholesterol, fasting glucose, alanine aminotransferase, aspartate aminotransferase, total bilirubin, hemoglobin, platelet count, leukocyte count were measured by an automated chemical analyzer (Olympus Au2700, First Chemical Ltd., Japan).

### Diagnosis of sarcopenia

According to the latest Asian Working Group for Sarcopenia (AWGS) 2019 criteria, sarcopenia was defined as low muscle mass plus low muscle strength and/or low physical performance [24]. Muscle mass was measured by bioelectrical impedance analysis (BIA) (Inbody S10; Inbody Korea Ltd., Korea). Appendicular skeletal muscle mass (ASM) was calculated as the sum of skeletal muscle in the arms and legs by using the formula: ASM = 0.193 × bodyweight + 0.107 × height − 4.157 × gender − 0.037 × age − 2.631 [24]. The height-adjusted appendicular skeletal muscle mass index (ASMI) was defined as ASM divided by height squared in meters (ASM/height^2^). Low muscle mass was defined as an ASMI of less than 7.0 kilogram (kg)/m^2^ in men and 5.7 kg/m^2^ in women [24]. Muscle strength was assessed by grip strength and measured using a dynamometer (CAMRY EH101, China). In standing position, the left and right handgrip strength was measured alternately three times and the maximum value was taken. Low handgrip strength was defined as <28 kg in men and <18 kg in women [24]. Usual gait speed on a 6-meter test was used as an objective measure of physical performance and a slow walking speed was defined as a walking speed less than 1 m/s [24]. The test was measured three times and the average value was calculated.

### Assessment of health-related quality of life (HRQoL)

The EuroQol 5-dimension 3-level (EQ-5D-3L) questionnaire was used for the HRQoL evaluation [25].The EQ-5D-3L descriptive system comprises five dimensions: mobility, self-care, usual activities, anxiety/depression, and pain/discomfort. Each dimension has three levels: no problems, some problems, and extreme problems. The health status was transformed into utility index based on the Chinese utility value integral system with the value range from -0.149 to 1.000 [26]. The utility score was 1.000 when all five dimensions were at the first level, which indicates a state of complete health. The utility score was -0.149 when all five dimensions were at the third level, which indicates the worst state of health.

### Assessment of socioeconomic status

The present study selected educational attainment, occupation before retirement, and monthly household income to measure the socioeconomic status of older adults as previously described [27]. Educational attainment is divided into the following four categories: illiteracy/ semi-illiteracy, primary school, middle school, and high school or college. Occupation before retirement is divided into the following four groups: (1) agricultural, forestry and fishery workers, (2) technicians and craft related trades workers, (3) clerical support workers, and (4) service and sales workers. Each respondent was asked to report the monthly household income in Chinese currency Yuan (CNY, ¥). We minimized the effect of cross-city economic disparities by subtracting the minimum monthly wage decreed by the Ministry of Human Resources and Social Security of China. This adjusted monthly household income was divided into three levels using quantile classification. The cutoff points were ¥2000 and ¥5000 (the exchange rate was approximately $1 USD = ¥6.54 CNY at the time of survey).

### Assessment of Lifestyle

The information of lifestyle was collected based on smoking, alcohol drinking, physical activity, and daily dietary habits. Smokers were defined as those who had smoked greater than 100 cigarettes in their life and who currently smoked cigarettes [28]. Regarding alcohol consumption, subjects were categorized as (1) non-drinker (never), (2) occasional drinker (≤ 1.43 g/day), and (3) moderate drinker (> 1.43 g/day) [29]. The information of daily dietary habits included tea, alcohol consumption, balanced diet, pickled food, salt intake, spicy frequency, skipping breakfast, fruit frequency, nut intake, and volume of drinking water was collected by using a dietary questionnaire as previously described [30]. The degree of salt intake was classified as low (<3 g/day), moderate (3-5 g/day), and high (5 g/day) as previous recommended [31]. The subjects who consumed spicy food at least once per week were defined as “regular consumers” [32]. The degree of water intake was classified as little (< 0.5 L/day), moderate (0.5-1.5 L/day), and much (> 1.5 L/day) as previous described [33].

### Statistical analysis

The normality of continuous variables was assessed using the Kolmogorov–Smirnov test. Continuous variables were reported as mean ± standard deviation (SD) or median and interquartile range (IQR). One-way ANOVA, Welch test, or chi-square test was applied to examine the differences among the three stages of sarcopenia (non-sarcopenia, normal sarcopenia, and severe sarcopenia). Univariate ordinary analysis and multivariate ordinary logistic regression analysis were taken to determine the variables that independently contributed to the presence of sarcopenia. Odds ratios (ORs) and 95% confidence intervals (CIs) were calculated. The correlation between variables and the score of EQ-5D-3L was calculated using Spearman correlation coefficient. Causal mediation models were constructed to find the potential mediating roles of age, body mass index, or waistline in the effect of HRQoL on sarcopenia risk according to the methods described previously [34, 35]. The causal mediation analysis was performed by using R Studio (version 3.6.3). Other analyses were conducted using SPSS 28.0 (IBM SPSS, Inc., USA). A two-sided *P* <0.05 was considered to be statistically significant.

## Results

### Characteristics of enrolled subjects

Demographic data of 2877 participants are shown in Table 1 according to the stages of sarcopenia. The prevalence of sarcopenia in this study was 20.7%. In terms of age groups, the prevalence of sarcopenia was 10.7%, 21.8%, and 45.7% in adults aged 60–69, 70–79, and ≥ 80 years, respectively. The prevalence of severe sarcopenia was 8.4%. The overall prevalence of sarcopenia was 23.5% in men and 17.8% in women. Patients with sarcopenia were older and had lower body mass index and waist circumference (*P* < 0.001). In addition, sarcopenic patients had higher proportion of widows/widowers, those living alone, hypertension, and diabetes (*P* < 0.01), as well as lower levels of triglycerides, alanine aminotransferase, hemoglobin, and higher level of high-density lipoprotein (*P* < 0.001).

**Table 1:** Characteristics of participants according to different stages of sarcopenia

	Non-sarcopenia *n=2291*	Normal-sarcopenia *n=354*	Severe-sarcopenia *n=242*	*P*
Assessment of sarcopenia				
ASMI (kg/m^2^)	7.16 (7.10–7.21)	5.90 (5.82–5.98)	5.72 (5.62–5.82)	**<0.001**
Grip strength (kg)	26.3 (26.0–26.7)	23.4 (22.7–24.0)	19.0 (18.4–19.7)	**<0.001**
gait speed (m/s)	1.06 (1.05–1.07)	1.00 (0.98–1.02)	0.83 (0.81–0.85)	**<0.001**
Demographics				
Age (years)	71.2 (71.0–71.4)	73.5 (72.9–74.1)	77.4 (76.6–78.1)	**<0.001**
Males, n (%)	1104 (48.2)	203 (52.2)	138 (57.0)	**<0.001**
Body mass index (kg/m^2^)	24.8 (24.7–25.0)	21.9 (21.6–22.2)	21.8 (21.4–22.3)	**<0.001**
Waist circumference (cm)	84.2 (83.8–84.6)	78.1 (77.2–79.1)	78.6 (77.4–79.8)	**<0.001**
Marriage status (%)				**0.001**
Married, n (%)	1941 (84.7)	280 (79.1)	180 (74.4)	
Unmarried, n (%)	40 (1.7)	11 (3.1)	6 (2.5)	
Divorce, n (%)	4 (0.2)	1 (0.3)	/	
Widowed, n (%)	306 (13.4)	62 (17.5)	56 (23.1)	
Residency				**<0.001**
Live alone, n (%)	344 (15.0)	74 (20.9)	51 (21.1)	
Live with spouse, n (%)	1428 (62.3)	191 (54.0)	108 (44.6)	
Live with children, n (%)	195 (8.5)	36 (10.2)	48 (20.0)	
Live with spouse and children, n (%)	324 (14.1)	53 (15.0)	35 (14.5)	
Income sources				**<0.001**
Pension, n (%)	69 (3.0)	12 (3.4)	4 (1.7)	
Child supply, n (%)	125 (5.5)	28 (7.9)	24 (9.9)	
Spouse supply, n (%)	49 (2.1)	6 (1.7)	2 (0.8)	
Government assistance, n (%)	30 (1.3)	10 (2.8)	15 (6.2)	
Social endowment insurance, n (%)	623 (27.2)	91 (25.7)	68 (28.1)	
Others, n (%)	80 (3.49)	11 (3.1)	5 (2.1)	
Any two items, n (%)	1315 (57.4)	196 (55.4)	124 (51.2)	
Payment methods for medical expenses				0.724
Basic medical insurance for urban residents, n (%)	20 (0.9)	4 (1.1)	1 (0.4)	
Basic medical insurance for urban employees, n (%)	52 (2.3)	4 (1.1)	5 (2.1)	
All at own expense, n (%)	3 (0.1)	1 (0.3)	/	
New rural cooperative medical care, n (%)	2216 (96.7)	345 (97.5)	236 (97.5)	
Comorbidities				
Hypertension, n (%)	889 (38.8)	99 (28.0)	86 (35.5)	**<0.001**
Diabetes mellitus, n (%)	252 (11.0)	20 (5.7)	24 (9.9)	**0.008**
Lab panel				
Total cholesterol (mmol/L)	4.78 (4.73–4.83)	4.80 (4.68–4.92)	4.70 (4.55–4.85)	0.544
Triglycerides (mmol/L)	1.62 (1.58–1.67)	1.36 (1.29–1.44)	1.57 (1.15–1.99)	**<0.001**
High-density lipoprotein (mmol/L)	1.48 (1.46–1.49)	1.56 (1.53–1.60)	1.56 (1.51–1.61)	**<0.001**
Low-density lipoprotein (mmol/L)	2.10 (2.07–2.12)	2.09 (2.02–2.16)	2.03 (1.95–2.11)	0.343
Fasting glucose (mmol/L)	5.82 (5.76–5.87)	5.66 (5.49–5.83)	5.68 (5.49–5.87)	0.073
Alanine aminotransferase (U/L)	19.6 (18.9–20.2)	15.7 (14.6–16.8)	15.9 (14.2–17.6)	**<0.001**
Aspartate aminotransferase (U/L)	25.3 (24.8–25.8)	24.5 (23.5–25.6)	26.0 (23.9–28.0)	0.320
Total bilirubin (μmol/L)	13.8 (13.6–14.1)	13.4 (12.8–14.0)	13.2 (12.4–14.0)	0.221
Hemoglobin (g/L)	129.9 (129.3–130.4)	127.7 (126.2–129.2)	125.3 (123.4–127.3)	**<0.001**
Platelet (×10^9^/L)	146.7 (144.6–148.7)	142.1 (137.2–147.0)	152.7 (145.1–160.2)	0.052
Leukocyte (×10^9^/L)	5.54 (5.48–5.60)	5.45 (5.28–5.61)	5.60 (5.37–5.83)	0.498

Continuous variables were reported as median with interquartile range

*ASMI* appendicular muscle mass index

### Association of HRQoL, socioeconomic status, and lifestyle with the risk of sarcopenia

Patients with sarcopenia had poor performance of HRQoL, lower levels of educational attainment and income, as well as more intake of salt, alcohol, and spicy food except for the anxiety/depression domain (Supplementary Table 1). As shown in Table 2, even after adjustment for potential confounders including body mass index, hypertension, diabetes, marriage status, and residency status, good HRQoL, as reflected high levels of EQ-5D-3L index score, was still associated with decreased risk of sarcopenia (adjusted OR = 0.85, 95%CI = 0.69-0.95, *P* = 0.034). Moreover, high levels of household income were also correlated with decreased risk of sarcopenia (adjust OR = 0.64, 95%CI = 0.50-0.83, *P* = 0.001 for those with ¥2000-5000 per month; adjusted OR = 0.74, 95%CI = 0.57-0.95, *P* = 0.019 for those with more than ¥5000 per month). Similar results were observed when using EQ-5D-3L index score (adjusted OR = 0.12, 95%CI = 0.03-0.46, *P* = 0.002) and income (adjusted OR = 0.99, 95%CI = 0.98-1.00, *P* = 0.042) as continuous variables. By contrast, no significant correlation between educational level and the risk of sarcopenia was observed in the present study. In addition, more consumption of spicy food (adjusted OR = 1.34, 95%CI = 1.09-1.81, *P* = 0.037) and occasional intake of alcohol (adjusted OR = 1.46, 95%CI = 1.07-2.00, *P* = 0.016 when compared to those never drinking) were associated with higher risk of sarcopenia, while less consumption of salt (adjusted OR = 0.71, 95%CI = 0.52-0.96, *P* = 0.026) was associated with lower risk of sarcopenia when compared to those intake high amount of salt. Interestingly, we also found that skipping breakfast occasionally was associated with decreased risk of sarcopenia when compared to those taking breakfast every day (adjusted OR = 0.37, 95%CI = 0.21-0.64, *P* < 0.001).

**Table 2:** Associations of health-related quality of life, socioeconomic status, and lifestyle with sarcopenia risk

	Number of subjects	Crude OR (95% CI)	*P*	*Adjusted* OR (95% CI)^a^	*P*
Waist circumference	2287	1.01 (1.00–1.03)	0.088	1.00 (0.98–1.03)	0.632
Body mass index	2287	0.74 (0.70–0.78)	**<0.001**	0.77 (0.73–0.80)	**<0.001**
EQ-5D-3L					
*Continuous*	2287	0.10 (0.03–0.38)	**<0.001**	0.12 (0.03–0.46)	**0.002**
*Categorical*	2287				
<1	1242	1	/	1	**/**
≥1	1645	0.87 (0.70–0.97)	**0.046**	0.85 (0.69–0.95)	**0.034**
Income					
*Continuous*^b^	2287	0.99 (0.98–1.00)	**0.033**	0.99 (0.98–1.00)	**0.042**
*Categorical*	2287				
*≤ ¥2000*	789	1	/	1	/
*¥2000–5000*	990	0.57 (0.44–0.74)	**<0.001**	0.64 (0.50–0.83)	**0.001**
*≥ ¥5000*	1108	0.77 (0.60–0.99)	**0.034**	0.74 (0.57–0.95)	**0.019**
Educational attainment					
Illiteracy/ Semi-illiteracy	1313	1	/	1	/
Primary school	973	1.37 (0.63–2.96)	0.412	1.10 (0.50–2.42)	0.820
Middle school	541	1.05 (0.48–2.28)	0.897	0.80 (0.36–1.74)	0.569
High school or college	60	0.89 (0.40–1.96)	0.756	0.81 (0.37–1.81)	0.615
Drinking frequency					
Non-drinker	2286	1	/	1	/
Occasional drinker	128	1.36 (1.00–1.85)	**0.038**	1.46 (1.07–2.00)	**0.016**
Moderate drinker	473	1.00 (0.55–1.81)	0.986	0.99 (0.55–1.77)	0.974
Spicy frequency					
Never	2407	1	/	1	/
Regular consumers	480	1.43(1.06–1.94)	**0.014**	1.34 (1.09–1.81)	**0.037**
Breakfast					
Everyday	2816	1	/	1	/
Skip occasionally	71	0.39 (0.23–0.68)	**<0.001**	0.37 (0.21–0.64)	**<0.001**
Salt intake					
High	342	1	/	1	/
Moderate	1914	0.65 (0.48–0.88)	**0.003**	0.71 (0.52–0.96)	**0.026**
Low	631	0.72 (0.51–1.02)	0.050	0.79 (0.56–1.12)	0.187
Nut intake					
Yes	360	1	/	1	/
No	2527	0.79 (0.56–1.11)	0.148	0.78 (0.56–1.09)	0.148
Water intake					
Much	948	1	/	1	/
Moderate	1612	1.14 (0.82–1.60)	0.414	1.09 (0.78–1.53)	0.610
Little	327	0.96 (0.70–1.32)	0.801	0.94 (0.69–1.29)	0.701

Drinking frequency: non-drinker (never); occasional drinker (≤ 1.43 g/day); moderate drinker (>1.43 g/day). Spicy frequency: the subjects who consumed spicy food at least once per week were defined as “regular consumers”. Salt intake: low (<3 g/day); moderate (3–5 g/day); high (5 g/day). Water intake: little (<0.5 L/day); moderate (0.5–1.5 L/day); much (>1.5 L/day)

*EQ-5D-3L*, Euro-QoL-Five Dimensions-three levels

^a^adjusted for age, sex, body mass index, hypertension, diabetes, marriage status, and residency status. For waist circumference, OR was calculated after adjustment for age, sex, hypertension, diabetes, marriage status, and residency status

^b^OR (95%CI) was calculated for every 1000-Yuan increment

### Potential mediators for the effect of HRQoL on the risk of sarcopenia

Spearman correlation analysis showed that EQ-5D-3L index score was negatively correlated with age (*r* = -0.10, *P* < 0.001), body mass index (*r* = -0.06, *P* =0.002), and waist circumference (*r* = -0.04, *P* =0.033), but positively correlated with household income (*r* =0.09, *P* <0.001). Considering there was no significant difference of household income between subjects with and without sarcopenia, we further performed causal mediation analysis to explore how much age, body mass index, and waist circumference might explain the effect of HRQoL on the risk of sarcopenia. As shown in Table 3, after adjustment for the potential confounders including sex, educational attainment, hypertension, diabetes, marriage status, and residency status, the estimated proportion that mediated the total effect of HRQoL on the risk of sarcopenia by age was 28.0% (β = -0.21, *P* < 0.001). By contrast, body mass index and waist circumference did not seem to be mediators of the effect of HRQoL on the risk of sarcopenia.

**Table 3:** Causal mediation analysis of age, body mass index, and waist circumference as potential mediators on the effect of health-related quality of life on the risk of sarcopenia

Mediators		Euro-QoL-Five Dimensions-three levels index score		
		Estimate^a^	95% CI	*P* value
Age	Total effect^b^	− 0.73	− 1.02 to − 0.44	**<0.001**
	Direct effect^c^	− 0.53	− 0.80 to − 0.25	**<0.001**
	Indirect effect^d^	− 0.21	− 0.30 to − 0.11	**<0.001**
Body mass index	Total effect^b^	− 0.53	− 0.80 to − 0.25	**<0.001**
	Direct effect^c^	− 0.59	− 0.85 to − 0.33	**<0.001**
	Indirect effect^d^	0.06	− 0.02 to 0.14	0.129
Waist circumference	Total effect^b^	− 0.53	− 0.80 to − 0.25	**<0.001**
	Direct effect^c^	− 0.57	− 0.83 to − 0.30	**<0.001**
	Indirect effect^d^	0.04	− 0.03 to 0.10	0.231

Results based on covariate-adjusted causal mediation analyses. Analysis was adjusted for sex, educational attainment, hypertension, diabetes, marriage status, and residency status. Estimates were calculated taking the non-sarcopenia group as reference

*CI*, Confidence Interval

^a^Bootstrapped β coefficients. Negative estimates indicate that age, body mass index, and waist circumference are associated with the risk of sarcopenia

^b^Effect of health-related quality of life on the risk of sarcopenia includes the mediator’s (age, body mass index, or waist circumference) effect

^c^Effect of health-related quality of life on the risk of sarcopenia excludes the mediator’s (age, body mass index, or waist circumference) effect

^d^Effect of health-related quality of life on the risk of sarcopenia through the mediators (age, body mass index, or waist circumference)

## Discussion

The main findings of the present study showed that poor quality of life, low income and unhealthy diet habits were associated with high risk of sarcopenia in a Chinese community-dwelling elderly population. Our results may therefore provide a valuable information and strategy for the screening of older adults with high risk of sarcopenia.

In the elderly, sociodemographic factors including lower educational and income level, as well as the lack of social support have been found to be associated with lower levels of HRQoL [36]. Patients with sarcopenia had higher proportion of illiteracy or semi-illiteracy but lower proportion of educational level of high school or college; however, no significant correlation between educational level and the risk of sarcopenia was observed in the present study. Interestingly, we here showed a significant association between lower household income and higher risk of sarcopenia. Subjects with lower incomes may have less ability to buy high-quality foods and get nutritional prescriptions to improve the malnutritional status, which is the common risk factor for sarcopenia [37]. These findings may, therefore, emphasize the importance of strong policies supporting nutritional intakes, especially in the lower-income populations.

Another interesting finding of our present study was that dietary habits also correlated with the risk of sarcopenia. We showed that more intake of spicy food was associated with increased risk of sarcopenia. Although capsaicin, the primary ingredient of chili peppers, has been suggested to have potential health benefits; however, its role in the different types of skeletal muscle remains unclear [38]. Considering that aging-related sarcopenia is mainly characterized by reduced type II muscle fibers, further studies will be needed to investigate the exact effect of spicy food on the development of sarcopenia. In addition, we found that less intake of salt was related to decreased risk of sarcopenia. Our results are consistent with a previous study showing that excessive salt intake can lead to the impairment of skeletal muscle performance [39]. It has been reported that excess sodium in skeletal muscle leads to a downregulation of expression of sodium-potassium-chloride symporter 1, which is involved in the differentiation of skeletal myoblast [40]. Importantly, sodium in the skeletal muscle accumulates more in older people than in younger people [41]. Further prospective study are needed to investigate the effects of different amount of salt intake on the risk of sarcopenia, especially in the older adults. Sufficient energy and protein intake is crucial for the synthesis and maintenance of muscle mass [42]. Breakfast is often considered as the most important meal of the day [43]. Skipping breakfast has been reported to be associated with a significant decrease in daily intake of protein and energy [44, 45], as well as lower fat-free mass in healthy young subjects [46]. By contrast, our results showed that older adults skipping breakfast had lower risk of sarcopenia when compared to those taking breakfast every day. The different age and health status of the subjects from these two studies may contribute to the contradictory results, since a large proportion of the older adults in our present study had comorbidities such as diabetes. Moreover, skipping breakfast has also been considered as a form of intermittent fasting that may trigger autophagy, which in turn plays a protective effect on aging and aging-related diseases [47]. However, this does not mean that we recommend skipping breakfast as a strategy for the prevention of sarcopenia, since skipping breakfast has been correlated with increased risk of cardiovascular diseases, diabetes, obesity, and all-cause mortalities [48–51]. Further, high-quality randomized controlled trials are needed to investigate whether skipping or consuming breakfast is beneficial for the prevention of sarcopenia.

The potential limitations of our present study should also be considered. First, the HRQoL assessments were based on self-reports, which may cause recall bias. Second, HRQoL was judged merely by generic questionnaires which may not sufficiently be able to capture the indistinct effects of the condition in the elderly. Third, the questionnaire Sarcopenia and Quality of Life (SarQoL) is the most popular specific HRQoL questionnaire for sarcopenia, the vast majority of items in which are directly related to muscle function [52]. Fourth, the sample of this community could not represent all Chinese older adults due to different communities would have different types and levels of income and education levels which may influence the quality of life. Fifth, the thresholds used for moderate drinking is relatively low, which may mask the association of alcohol consumption with sarcopenia. Further studies with detailed information of alcohol consumption patterns and amounts are needed to demonstrate the dose–response relationship between alcohol consumption and the risk of sarcopenia. Finally, the cross-sectional study design does not permit to exclude the possibility of reverse causality. Therefore, further prospective cohort studies with diverse regions will be needed to corroborate our present findings.

## Conclusions

In summary, our findings demonstrate that low levels of HRQoL and household income, more intake of salt and spicy food, and occasional intake of alcohol are correlated with higher risk of sarcopenia, while skipping breakfast occasionally is associated with lower risk of sarcopenia in a Chinese population of older adults. Therefore, subjects with low levels of health quality and low income should be paid more attention for the screening and prevention of sarcopenia. In addition, a low-salt and less spicy diet might be healthy for the maintenance of skeletal muscle in the elderly. Nevertheless, further studies are needed to confirm the results of our study and explore the potential underlying mechanism for the association of lifestyle and quality of life with sarcopenia.

### Supplementary Information

Below is the link to the electronic supplementary material.

Supplementary file1 (DOCX 27 KB)

## Data Availability

The datasets generated during and/or analyzed during the current study are available from the corresponding author on reasonable request.
