# Supplementary material for: Quality of life, household income, and dietary habits are associated with the risk of sarcopenia among the Chinese elderly
Source: Aging Clin Exp Res. 2024 Feb 9;36(1):29. doi: 10.1007/s40520-023-02656-9 (PMC10857955; doi:10.1007/s40520-023-02656-9)
Supplement: Supplementary file 1 — Supplementary file1 (DOCX 27 KB) [file 40520_2023_2656_MOESM1_ESM.docx]

**The status of sarcopenia according to the level of HRQoL**

Regarding the items of EQ-5D-3L, patients with sarcopenia had poor performance of HRQoL, except for the Anxiety/Depression domain (**Supplementary Table 1**). By contrast, individuals without sarcopenia had higher levels EQ-5D-3L index score than those with sarcopenia, showing that the EQ-5D-3L index score decreased as the severity of sarcopenia increased (*P* for trend <0.01) (**Supplementary Table 1**).

**The status of sarcopenia according to the level of socioeconomic status**

Individuals without sarcopenia had higher levels of educational attainment and income than those with sarcopenia (*P* for trend <0.01) (**Supplementary Table 1**). Among different educational levels, the illiteracy or semi-illiteracy participants had the highest prevalence of sarcopenia, while those attaining middle school or higher school displayed lower prevalence of sarcopenia. No significant difference of the types of occupation was observed between subjects with and without sarcopenia.

**The status of sarcopenia according to the different lifestyles**

Participants with sarcopenia consumed more salt, alcohol, and spicy food than those without sarcopenia (*P* <0.05). By contrast, participants with sarcopenia had less intake of breakfast, water, and nuts when compared to those without sarcopenia (*P* <0.05) (**Supplementary Table 1**).

**Supplementary Table 1.** Health related quality of life, socioeconomic status and lifestyle status in subjects with non-sarcopenia, normal-sarcopenia and severe-sarcopenia.

|  | **Non-sarcopenia**  ***n=2291*** | **Normal-sarcopenia**  ***n=354*** | **Severe-sarcopenia**  ***n=242*** | ***P*** |
| --- | --- | --- | --- | --- |
| **HRQoL** |  |  |  |  |
| EQ-5D-3L descriptive system |  |  |  |  |
| Mobility, Yes, n=63 | 2251 (98.2) | 346 (97.7) | 227 (93.8) | **<0.001** |
| Self-care, Yes, n=2869 | 2282 (99.6) | 350 (98.9) | 237 (97.9) | **0.003** |
| Usual activities, No problems, n=2794 | 2241 (97.8) | 335 (94.6) | 218 (90.1) | **<0.001** |
| Anxiety/Depression, No, n=2852 | 2267 (99.0) | 348 (98.3) | 237 (97.9) | 0.262 |
| Pain/discomfort, No, n=1701 | 1367 (59.7) | 212 (59.9) | 122 (50.4) | **0.047** |
| EQ-5D-3L VAS (0-100) | 75.442 (75.024-75.860) | 74.537 (73.335-75.738) | 71.236 (69.666-72.805) | **<0.001** |
| EQ-5D-3L index score value | 0.941 (0.938-0.943) | 0.937 (0.929-0.945) | 0.915 (0.903-0.927) | **<0.001** |
| **SES** |  |  |  |  |
| Educational attainment |  |  |  | **0.009** |
| Illiteracy/ Semi-illiteracy, n (%) | 1012 (44.2) | 173 (48.9) | 128 (52.9) |  |
| Primary school, n (%) | 777 (33.9) | 110 (31.1) | 86 (35.5) |  |
| Middle school, n (%) | 452 (19.7) | 64 (18.1) | 25 (10.3) |  |
| High school or college, n (%) | 50 (2.2) | 7 (2.0) | 3 (1.2) |  |
| Occupation |  |  |  | 0.402 |
| Agricultural, forestry and fishery workers, n (%) | 56 (2.4) | 7 (2.0) | 4 (1.7) |  |
| Technicians and craft related trades workers, n (%) | 2223 (97.0) | 345 (97.5) | 236 (97.5) |  |
| Clerical support workers, n (%) | 6 (0.3) | / | 2 (0.8) |  |
| Service and sales workers, n (%) | 6 (0.3) | 2 (0.6) | / |  |
| Income, ¥ | 7246.118 (6758.478 - 7733.757) | 6371.893 (5110.949 - 7632.836) | 4403.256 (3339.309 - 5467.204) | **<0.001** |
| **Lifestyle** |  |  |  |  |
| Smoking |  |  |  | 0.900 |
| No, n (%) | 1818 (79.4) | 275 (77.6) | 195 (80.8) |  |
| Yes, n (%) | 447 (19.5) | 75 (21.3) | 44 (18.0) |  |
| Not now, n (%) | 26 (1.1) | 4 (1.1) | 3 (1.3) |  |
| Drinking frequency |  |  |  | **0.008** |
| Non-drinker, n (%) | 1964 (85.7) | 297 (83.9) | 189 (78.1) |  |
| Occasional drinker, n (%) | 263 (11.5) | 44 (12.4) | 42 (17.4) |  |
| Moderate drinker, n (%) | 64 (2.8) | 13 (3.7) | 11 (4.5) |  |
| Physical activity |  |  |  | 0.146 |
| No, n (%) | 1905 (83.2) | 309 (87.3) | 202 (83.6) |  |
| Yes, n (%) | 386 (16.8) | 45 (12.7) | 40 (16.4) |  |
| Tea |  |  |  | 0.057 |
| No, n (%) | 1951 (85.2) | 317 (89.5) | 206 (85.1) |  |
| Yes, n (%) | 440 (14.8) | 37 (10.5) | 36 (14.9) |  |
| Diet |  |  |  | 0.415 |
| Meaty diet, n (%) | 41 (1.8) | 8 (2.2) | 5 (2.1)) |  |
| Balanced diet, n (%) | 1561 (68.1) | 233 (65.8) | 151 (62.5) |  |
| Plant-based diet, n(%) | 689 (30.1) | 113 (32.0) | 86 (35.4) |  |
| Salt intake |  |  |  | **0.026** |
| Moderate, n (%) | 1548 (67.5) | 221 (62.6) | 145 (60.0) |  |
| Low, n (%) | 494 (21.6) | 79 (22.3) | 58 (24.0) |  |
| High, n (%) | 249 (10.9) | 54 (15.1) | 39 (16.0) |  |
| Spicy frequency |  |  |  | **0.028** |
| Never, n (%) | 1979 (86.4) | 309 (87.3) | 200 (82.6) |  |
| Twice per week, n (%) | 312 (13.6) | 45 (12.7) | 42 (17.4) |  |
| Breakfast |  |  |  | **0.008** |
| Every day, n (%) | 2176 (95.0) | 340 (96.0) | 237 (98.0) |  |
| Skip occasionally, n (%) | 115 (5.0) | 14 (4.0) | 5 (2.0) |  |
| Pickled food (3-5 times per week) |  |  |  | 0.626 |
| Yes, n (%) | 658 (28.7) | 99 (28.0) | 76 (31.4) |  |
| No, n (%) | 1633 (71.3) | 255 (72.0) | 166 (68.6) |  |
| Fruit intake (3-5 times per week) |  |  |  | 0.270 |
| Yes, n (%) | 436 (19.0) | 55 (15.5) | 48 (19.8) |  |
| No, n (%) | 1855 (81.0) | 299 (84.5) | 194 (80.2) |  |
| Nut intake (3-5 times per week) |  |  |  | **0.028** |
| Yes, n (%) | 304 (13.3) | 37 (10.5) | 19 (7.8) |  |
| No, n (%) | 1987 (86.7) | 317 (89.5) | 223 (92.2) |  |
| Water intake |  |  |  | **0.006** |
| Few, n (%) | 239 (10.4) | 48 (13.6) | 40 (16.5) |  |
| Moderate, n (%) | 1297 (56.6) | 180 (50.8) | 135 (55.8) |  |
| Much, n (%) | 755 (33.0) | 126 (35.6) | 67 (27.7) |  |

SES, Socioeconomic status; HRQoL, Health related quality of life; EQ-5D-3L, Euro-QoL-Five Dimensions-three levels; VAS, Visual analogue scale.
